# SseL Deubiquitinates RPS3 to Inhibit Its Nuclear Translocation

**DOI:** 10.3390/pathogens7040086

**Published:** 2018-11-07

**Authors:** Miaomiao Wu, Samir El Qaidi, Philip R. Hardwidge

**Affiliations:** Department of Diagnostic Medicine/Pathobiology, Kansas State University, Manhattan, KS 66506, USA; miaomiaowu@k-state.edu (M.W.); elqaidi@ksu.edu (S.E.Q.)

**Keywords:** effector, nuclear translocation, RSP3, SseL, ubiquitination

## Abstract

Many Gram-negative bacterial pathogens use type III secretion systems to deliver virulence proteins (effectors) into host cells to counteract innate immunity. The ribosomal protein S3 (RPS3) guides NF-κB subunits to specific κB sites and plays an important role in the innate response to bacterial infection. Two *E. coli* effectors inhibit RPS3 nuclear translocation. NleH1 inhibits RPS3 phosphorylation by IKK-β, an essential aspect of the RPS3 nuclear translocation process. NleC proteolysis of p65 generates an N-terminal p65 fragment that competes for full-length p65 binding to RPS3, thus also inhibiting RPS3 nuclear translocation. Thus, *E. coli* has multiple mechanisms by which to block RPS3-mediated transcriptional activation. With this in mind, we considered whether other enteric pathogens also encode T3SS effectors that impact this important host regulatory pathway. Here we report that the *Salmonella* Secreted Effector L (SseL), which was previously shown to function as a deubiquitinase and inhibit NF-κB signaling, also inhibits RPS3 nuclear translocation by deubiquitinating this important host transcriptional co-factor. RPS3 deubiquitination by SseL was restricted to K63-linkages and mutating the active-site cysteine of SseL abolished its ability to deubiquitinate and subsequently inhibit RPS3 nuclear translocation. Thus, *Salmonella* also encodes at least one T3SS effector that alters RPS3 activities in the host nucleus.

## 1. Introduction

Gram-negative bacteria export virulence proteins (effectors) into host cells using a type three secretion system (T3SS) [1]. Effectors bind and/or post-translationally modify host proteins, preventing the host from generating inflammatory responses to the pathogen. Enterohemorrhagic *E. coli* (EHEC) is an attaching/effacing (A/E) pathogen that causes hemorrhagic colitis and pediatric renal failure in humans [2]. The EHEC T3SS and some effectors are encoded on a pathogenicity island termed the locus of enterocyte effacement (LEE) [3]. Most of the effectors encoded within the LEE are referred to as *E. coli* secreted proteins (Esps), while effectors encoded outside the LEE are referred to as non-LEE encoded effectors (Nles). *Salmonella enterica* serovars encode multiple T3SSs; T3SS-1 is encoded on the *Salmonella* pathogenicity island (SPI-1) and delivers effectors across the host cell plasma membrane to trigger actin rearrangements that contribute to bacterial invasion [4]. T3SS-2 is encoded on SPI-2 and translocates virulence proteins from the *Salmonella* containing vacuole (SCV) into the host cell cytoplasm [5].

The recognition of bacterial pathogens by host cells triggers multiple signaling pathways to induce host inflammatory responses [6], many of which are regulated by the nuclear factor kappa-light-chain-enhancer of activated B cells (NF-κB). The ribosomal protein S3 (RPS3) is a component of the eukaryotic 40S small ribosomal subunit and plays multifunctional roles in DNA repair and apoptosis [7,8]. RPS3 possesses an endonuclease activity that mediates some DNA repair processes [9]. Knockdown of RPS3 protects cells from genotoxic stress after hydrogen peroxide treatment [10]. RPS3 can be phosphorylated by PKCδ, leading to its mobilization in the nucleus to repair damaged DNA [11]. RPS3 is one of a large number of ribosomal proteins that have extraribosomal functions [12]. Many of these functions are related to tumorigenesis [13], immune signaling [14], and cell development [15].

RPS3 functions as a “specifier” component in NF-κB complexes [16]. RPS3 guides NF-κB to specific κB sites by increasing the affinity of the NF-κB p65 subunit for target gene promoters [16]. RPS3 associates with p65 in the inhibitory p65-p50-IκBα complex in the cytoplasm of resting cells [16]. Activation of NF-κB is initiated by external stimuli that activate the IκB kinase (IKK) complex. Activated IKKβ phosphorylates IκBα, leading to its subsequent ubiquitination and degradation, which allows for p65 and p50 nuclear translocation [17]. IKKβ also phosphorylates RPS3 on Ser209, enhancing its association with importin-α and mediating RPS3 nuclear translocation [18].

Two EHEC effectors disrupt the activation of the innate immune system of intestinal epithelial cells by inhibiting RPS3 nuclear translocation. NleH1 binds to RPS3 [19] and inhibits its phosphorylation by IKK-β [18]. NleC proteolysis of p65 generates an N-terminal p65 fragment that competes for full-length p65 binding to RPS3, thus inhibiting RPS3 nuclear translocation [20]. Thus, *E. coli* has multiple mechanisms by which to block RPS3-mediated transcriptional activation. With this in mind, we considered whether other enteric pathogens also encode T3SS effectors that affect this important host regulatory pathway. Here we report that the *Salmonella* Secreted Effector L (SseL), which has been previously shown to inhibit NF-κB signaling [21] and function as a deubiquitinase [22], also inhibits RPS3 nuclear translocation.

## 2. Results

### 2.1. SseL Reduces the Nuclear Abundance of RPS3

**Figure 1:**
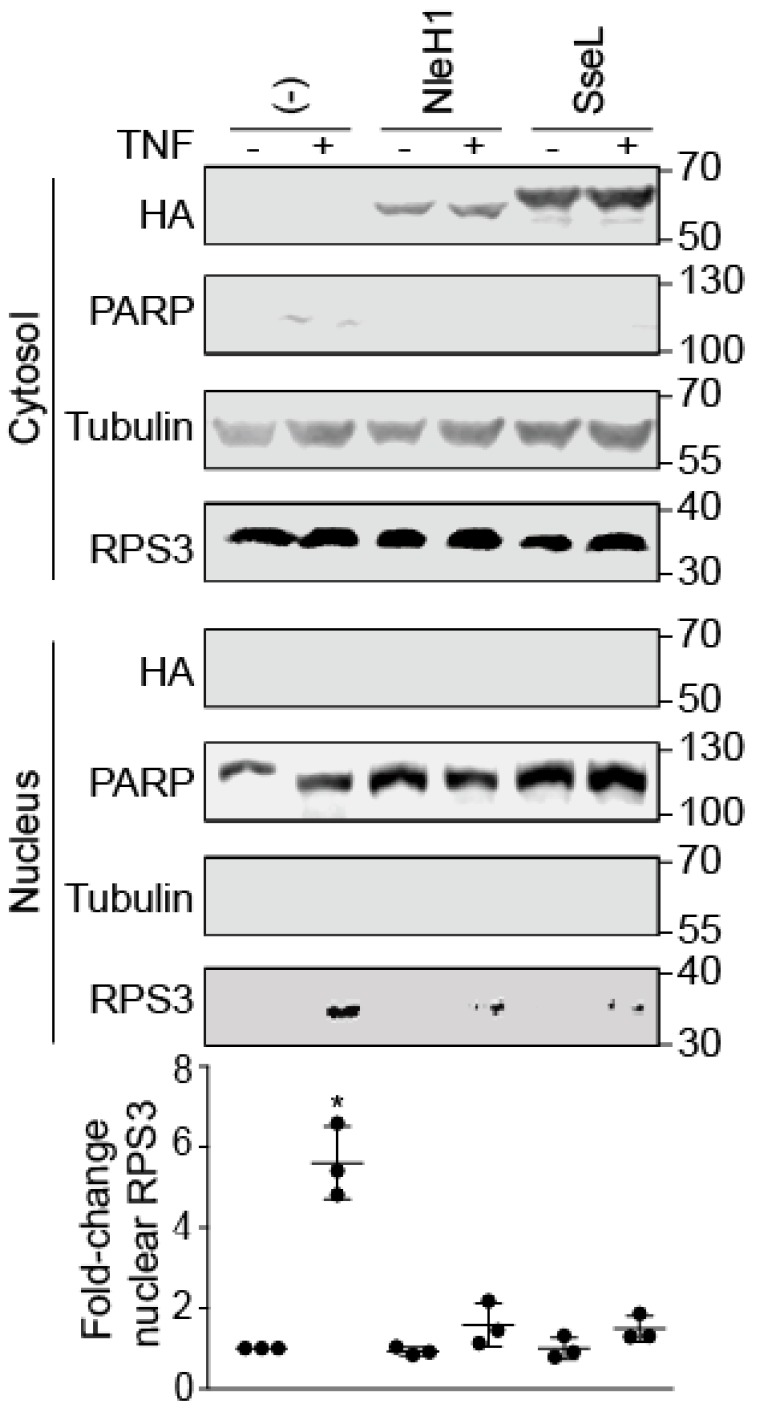
SseL inhibits RPS3 nuclear translocation. HEK293 cells were transfected with the indicated plasmids and treated with TNF-α (50 ng/mL, 30 min) 24 h later. The cells were lysed, separated into nuclear and cytosolic extracts, and subjected to immunoblotting using the indicated antibodies. Poly(ADP-ribose) polymerase (PARP) and tubulin were used to normalize nuclear and cytosolic protein concentrations, respectively. RPS3 quantification data (n = 3) are shown as means ± S.E after normalization to nuclear PARP abundance. Asterisks indicate significantly different protein abundance as compared with the TNF-α control (*p* < 0.05, Dunn’s multiple comparisons test). Representative images from three independent experiments are shown. We note a small difference in PARP mobility between untreated samples and samples that were either transfected or treated with TNF-α.

RPS3 translocates to the nucleus after stimulation with human tumor necrosis factor-α (TNF-α) [16]. The *Salmonella* T3SS effector protein SseL impairs IκBα ubiquitination and degradation [6]. To determine whether SseL also inhibits RPS3 nuclear translocation, we quantified the relative abundance of nuclear vs. cytoplasmic RPS3 in HEK293 cells in the presence or absence of transfected SseL-HA after TNF stimulation. As expected, the nuclear abundance of RPS3 significantly increased after TNF treatment. EHEC NleH1, a positive control, significantly decreased RPS3 nuclear translocation. The nuclear abundance of RPS3 was reduced in nuclear fractions containing SseL, while cytoplasmic RPS3 concentrations were unchanged. SseL was detected only in the cytoplasm. Quantitative analysis of RPS3 abundance revealed that SseL significantly reduced the relative abundance of nuclear RPS3 after TNF stimulus (Figure 1).

### 2.2. SseL Binds to RPS3

**Figure 2:**
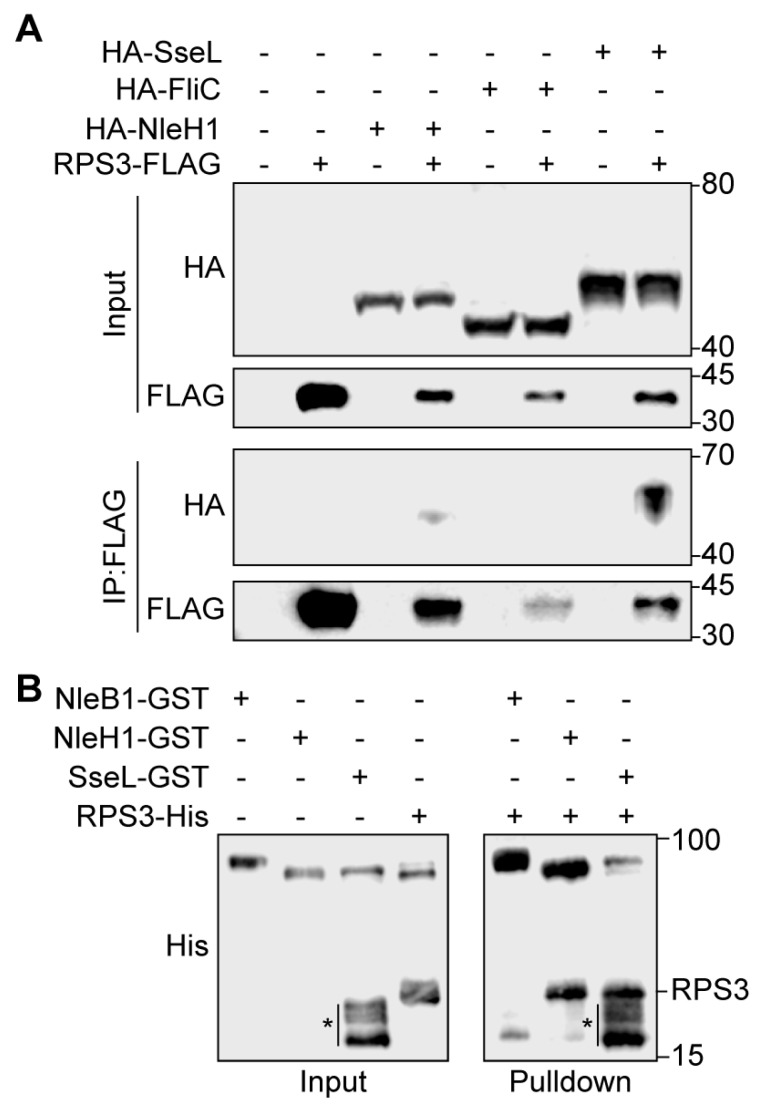
SseL binds to the host ribosomal protein S3 (RPS3). (**A**) Co-immunoprecipitation of SseL-HA from HEK293 cells with FLAG-RPS3. HEK293 cells were transfected with the indicated plasmids. Cell lysates were immunoprecipitated using anti-FLAG M2 gel and immunoblotted for FLAG and HA. (**B**) Pulldown assay to detect binding between SseL and RPS3. His-RPS3 was incubated with GST-SseL and subjected to GST pulldown assay using glutathione-sepharose beads (GE Healthcare). Protein complexes were eluted with 10 mM reduced glutathione followed by 10% SDS-PAGE analysis. GST-NleB was used as a negative control, and GST-NleH1 was used as positive control. Representative images from three independent experiments are shown. Asterisk denotes non-specific bands generated from the breakdown of the SseL-GST fusion protein.

To determine whether SseL interacts with RPS3 in mammalian cells, we performed co-immunoprecipitation experiments. After co-transfecting either FliC-HA (as a negative control), NleH1-HA (as a positive control), or SseL-HA with FLAG-RPS3, cell lysates were immunoprecipitated with anti-FLAG M2 beads and subsequently immunoblotted. NleH1-HA and SseL-HA, but not FliC-HA, interacted with FLAG-RPS3 (Figure 2A). To determine whether SseL binds directly to RPS3, we conducted GST pulldown assays. As NleH1 is known to bind to RPS3 [19], we used GST-NleH1 as a positive control and GST-NleB1 as a negative control [23]. Purified GST-NleH1, GST-NleB1, and GST-SseL were immobilized on GST beads and incubated with RPS3-His. RPS3 was enriched in the NleH1 and SseL pulldown samples, as compared to the negative control (Figure 2B). Thus, SseL interacts with RPS3 in mammalian cells and binds directly to RPS3 in vitro.

### 2.3. SseL Deubiquitinates RPS3

**Figure 3:**
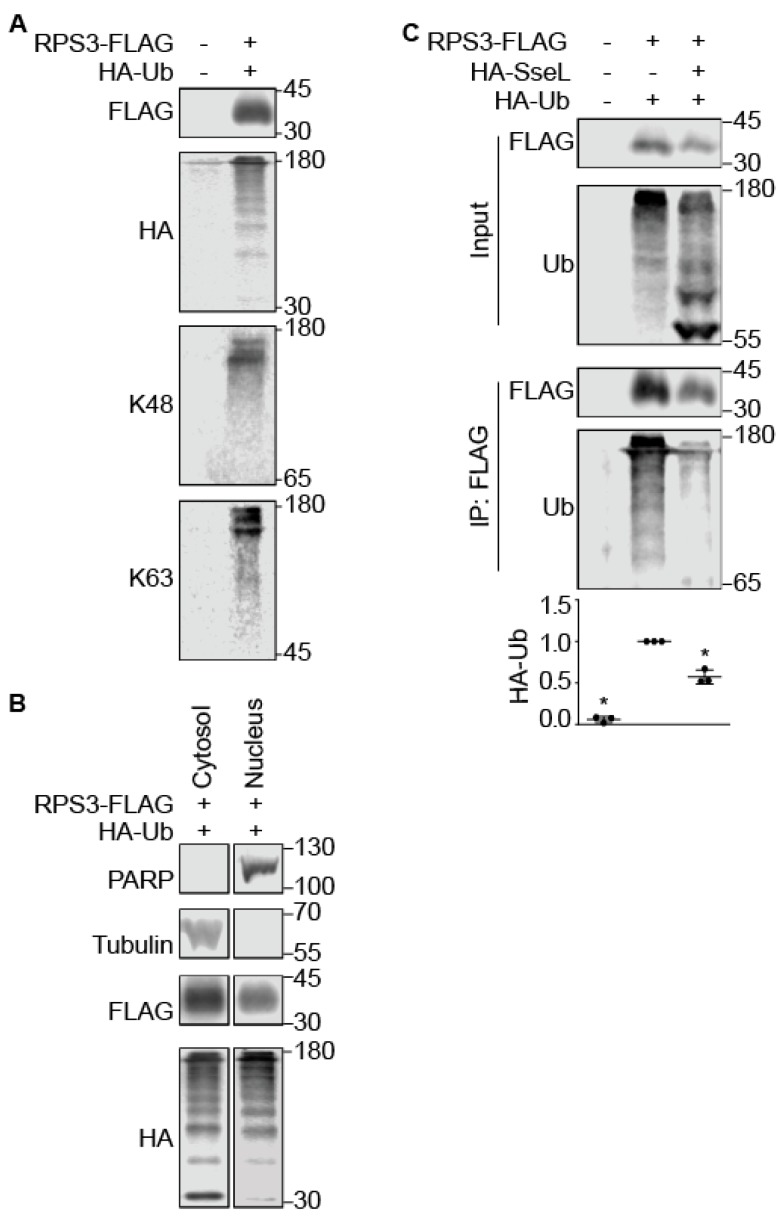
SseL deubiquitinates RPS3. (**A**) RPS3 ubiquitination. HEK293 cells were co-transfected with FLAG-RPS3 and HA-ubiquitin plasmids. After 24 h, cell lysates were immunoprecipitated using anti-FLAG M2 gel and immunoblotted for FLAG, HA, K48-Ub, and K63-Ub. (**B**) Nuclear RPS3 is ubiquitinated. HEK293 cells were co-transfected with FLAG-RPS3 and HA-ubiquitin plasmids. After 24 h, cells were stimulated with TNF-α (50 ng/mL, 30 min) and then lysed, separated into nuclear and cytosolic extracts. These samples were then immunoprecipitated using anti-FLAG M2 gel and then used in immunoblotting experiments. (**C**) SseL deubiquitinates RPS3. HEK293 cells were transfected with the indicated plasmids. After 24 h, cell lysates were immunoprecipitated using anti-FLAG M2 gel and immunoblotted. Quantification (n = 3) of the fold-change in ubiquitin signal intensity after normalization with FLAG-RPS3 intensity is shown as mean ± S.E. Asterisks indicate significantly different protein abundance as compared with ubiquitinated RPS3 control group (*p* < 0.05, Dunn’s multiple comparisons test). Representative images from three independent experiments are shown.

SseL is a deubiquitinase (DUB) that induces a delayed cytotoxic effect in *Salmonella-*infected macrophages [22] by impairing IκBα ubiquitination and degradation [21]. IKKβ-mediated phosphorylation of RPS3 S209 is a prerequisite for RPS3 nuclear translocation [18]. We hypothesized that the deubiquitinase activity of SseL might be required for its ability to inhibit RPS3 nuclear translocation. Ubiquitination of RPS3 has been recently demonstrated to be important for ribosome quality control [24,25], but the role of ubiquitination in potentially regulating RPS3 nuclear translocation has been less extensively studied. We first determined that RPS3 is ubiquitinated and that such ubiquitination can occur using both K48 and K63 isopeptide linkages (Figure 3A). Ubiquitinated RPS3 was detected in the nucleus as well as the cytoplasm, as shown by performing nuclear fractionation assays (Figure 3B). Co-transfecting SseL significantly decreased the extent of RPS3 ubiquitination (Figure 3C).

### 2.4. SseL DUB Activity Is Important to Inhibiting RPS3 Nuclear Translocation

**Figure 4:**
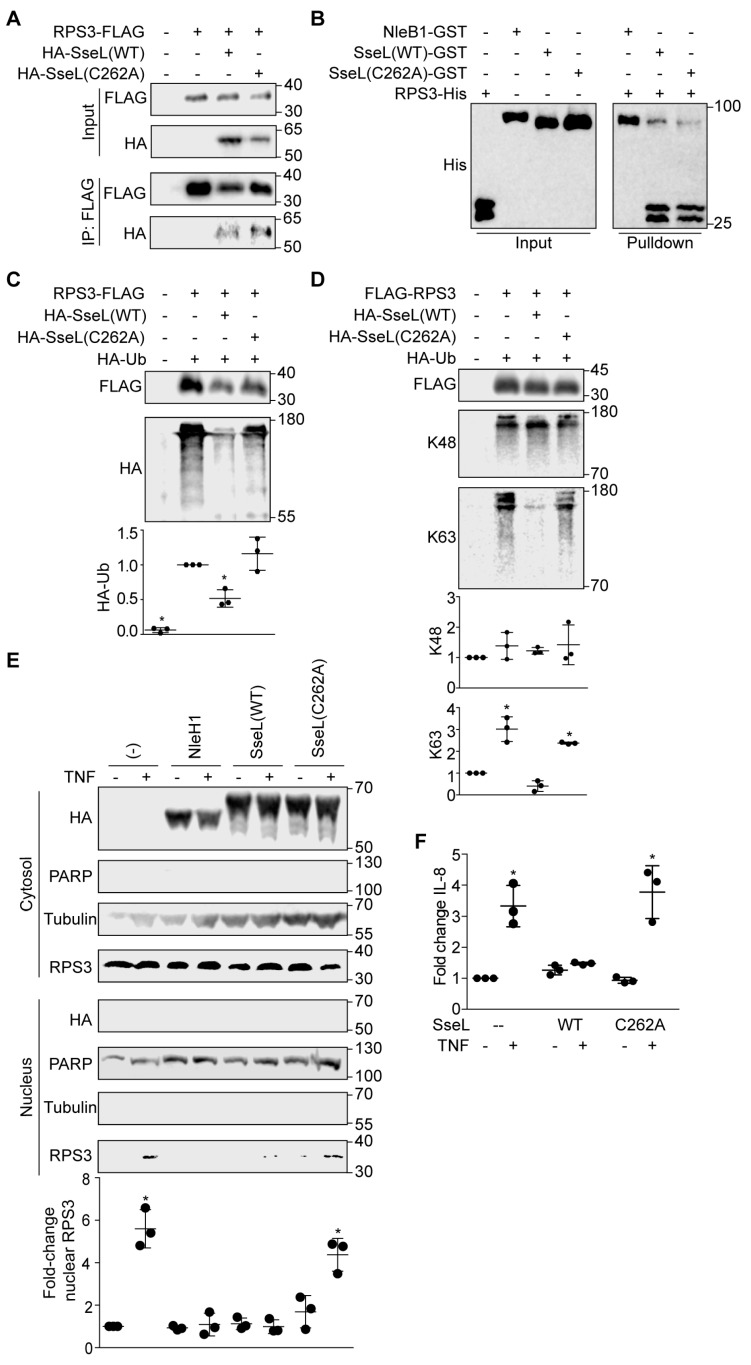
SseL DUB activity blocks RPS3 nuclear translocation. (**A**) Co-immunoprecipitation of SseL WT-HA and SseL(C262A)-HA with FLAG-RPS3. HEK293 cells were transfected with the indicated plasmids, immunoprecipitated using anti-FLAG M2 gel, and immunoblotted for FLAG and HA. (**B**) Pulldown assay to detect binding between SseL(C262A) and RPS3. His-RPS3 was incubated with GST-SseL WT or GST-SseL(C262A) and subjected to GST pulldown assay using glutathione-sepharose beads. Protein complexes were eluted with 10 mM reduced glutathione followed by 10% SDS-PAGE analysis. GST-NleB1 was used as a negative control. (**C**) SseL(C262A) fails to deubiquitinate RPS3. HEK293 cells were transfected with the indicated plasmids, immunoprecipitated using anti-FLAG M2 gel, and immunoblotted for FLAG and HA. Quantification (n = 3) of the fold-change in ubiquitin signal intensity after normalization with FLAG-RPS3 intensity is shown as mean ± S.E. Asterisks indicate significantly different protein abundance as compared with ubiquitinated RPS3 control group (*p* < 0.05, Dunn’s multiple comparisons test). (**D**) SseL DUB activity on RPS3 is specific to K63 linkages. HEK293 cells were transfected with the indicated plasmids, immunoprecipitated using anti-FLAG M2 gel, and immunoblotted for FLAG, HA, K48-Ub, and K63-Ub. Quantification (n = 3) of the fold-change in ubiquitin signal intensity after normalization with FLAG-RPS3 intensity is shown as mean ± S.E. Asterisks indicate significantly different protein abundance as compared with ubiquitinated RPS3 control group (*p* < 0.05, Dunn’s multiple comparisons test). (**E**) SseL C262A fails to inhibit RPS3 nuclear translocation. HEK293 cells were transfected with the indicated plasmids. After 24 h, cells were stimulated with TNF-α (50 ng/mL, 30 min) and then lysed, separated into nuclear and cytosolic extracts, and used in immunoblotting experiments. RPS3 quantification data (n = 3) are shown as means ± S.E after normalization to nuclear PARP abundance. Asterisks indicate significantly different protein abundance as compared with the TNF-α control (*p* < 0.05*,* Dunn’s multiple comparisons test). We note a small difference in PARP mobility between untreated samples and samples that were either transfected or treated with TNF-α. Representative images from three independent experiments are shown. (**F**) SseL inhibits IL-8 expression. HEK293 cells were transfected with either SseL-WT or SseL(C262A) and then stimulated with 50 ng/mL TNF-α for 30 min. IL-8 expression was quantified using RT-PCR, and data were normalized to beta-actin expression.

Mutating SseL C262 abolishes its ability to hydrolyze ubiquitin [22]. To determine whether SseL C262 is required for its interaction with RPS3, we mutated C262 into alanine and performed co-immunoprecipitation and GST pulldown assays. We observed that both SseL wild-type and SseL C262A interacted with RPS3 (Figure 4A), and they both bound directly to RPS3 (Figure 4B), indicating that this residue is not essential for SseL binding to RPS3. The SseL C262A mutant, in contrast to SseL wild-type, showed a reduced ability to deubiquitinate RPS3 after co-transfection (Figure 4C). RPS3 deubiquitination by SseL was restricted to K63 linkages (Figure 4D).

**Figure 5:**
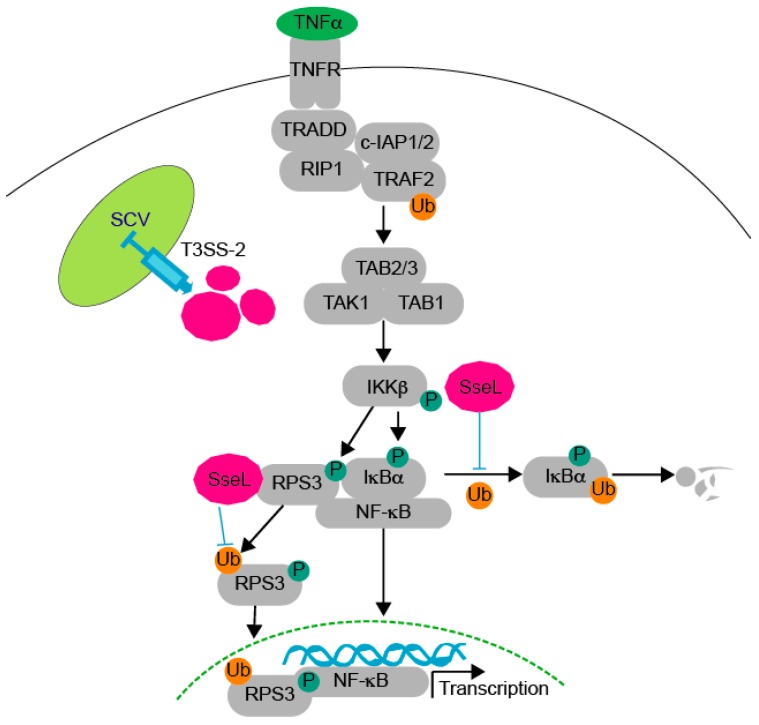
Working model. After TNF-α stimulation through the tumor necrosis factor receptor (TNFR), transforming growth factor beta-activated kinase 1 (TAK1) activation is induced, promoting IKKβ activation, which then phosphorylates both IκBα and RPS3. During *Salmonella* infection, SseL is translocated into host cells through the T3SS-2 and then interacts with both IκBα and RPS3. SseL impairs IκBα ubiquitination and degradation, thus limiting host NF-κB pathway activation and promoting bacterial pathogen colonization [6]. SseL binds and deubiquitinates RPS3, resulting in reduced RPS3 nuclear translocation.

To determine whether SseL DUB activity is important for inhibiting RPS3 nuclear translocation, we transfected HEK293T cells with WT SseL-HA or SseL(C262A)-HA and evaluated the relative abundance of nuclear vs. cytoplasmic RPS3. The nuclear abundance of RPS3 was unchanged in the nuclear fraction of cells transfected with SseL(C262A), but was significantly inhibited in cells transfected with SseL wild-type (Figure 4E). To evaluate whether SseL alters RPS3/NF-κB-dependent gene expression, we performed quantitative RT-PCR experiments to assess whether SseL reduced the ability of TNF-α to activate the expression of IL-8, a gene that is dependent upon RPS3 for maximal expression [19]. TNF-α induced IL-8 expression was significantly inhibited by WT SseL but not by SseL(C262A) (Figure 4F), showing the biological significance of how the SseL-mediated reduction in RPS3 nuclear abundance affects host transcriptional responses associated with innate immunity. Taken together, our data suggest that SseL functions as a DUB that inhibits RPS3 nuclear translocation by deubiquitinating RPS3 (Figure 5).

## 3. Discussion

Here we further examined the mechanism of RPS3 nuclear translocation and gained novel insights into the importance of RPS3 ubiquitination in this process. We provide evidence that the *Salmonella* effector SseL binds to RPS3 and functions as a DUB to inhibit its nuclear translocation. RPS3 was identified as a “specifier” component in NF-κB complexes [26]. RPS3 guides NF-κB to specific κB sites by increasing the affinity of the NF-κB p65 subunit for target gene promoters [16].

Evidence is emerging that some ribosomal proteins have extraribosomal functions that affect NF-κB signaling. The ribosomal protein rpL3 stabilizes IκBα to inhibit p65 nuclear translocation in p53-mutated cells, thus reducing IL-8 production [27]. TNF-induced synthesis of H_2_S sulfhydrates the C38 residue of the NF-κB p65 subunit and promotes p65 association with RPS3 to enhance the expression of cytoprotective genes [28]. Human RPS3 contains 20 lysine residues that are potential ubiquitination sites [29,30,31]. The unfolded protein response triggers ubiquitination of RPS3 K214 [32]. Regulatory RPS3 ubiquitination catalyzed by ZNF598 plays a pivotal role in regulating mammalian ribosome-associated quality control pathways [24,25,33]. RPS3 was also shown to interact with p53 [34], but its mono-ubiquitination is independent of p53 [24]. 

Non-ribosomal RPS3 is protected from ubiquitination and proteasome-dependent degradation by interacting with Heat shock protein 90 (Hsp90), which helps retain the function and biogenesis of the ribosome [35]. There is little information available concerning the role of RPS3 ubiquitination in its nuclear translocation, although it is known that nuclear RPS3 can be ubiquitinated by ring finger protein 138 (RNF138) [8]. This ubiquitination leads to RPS3 degradation and affects radioresistance [8]. As no active E3 ligase that is specific to RPS3 is commercially available, we were unable to perform experiments to detect RPS3 ubiquitination and deubiquitination in vitro*.*

Two *E. coli* effectors inhibit RPS3 nuclear translocation; NleH1 inhibits RPS3 phosphorylation by IKK-β an essential aspect of the RPS3 nuclear translocation process [19]. NleC proteolysis of p65 generates an N-terminal p65 fragment that competes for full-length p65 binding to RPS3, thus also inhibiting RPS3 nuclear translocation [20]. We examined whether *Salmonella* effectors also target this pathway, and, by using nuclear fractionation and transfection experiments, we observed a significant reduction in nuclear RPS3 abundance by expressing SseL (Figure 1). SseL interacted with RPS3 in mammalian cells and bound directly to RPS3 in vitro (Figure 2). SseL deubiquitinated RPS3 in transfected mammalian cells (Figure 3). However, we were unable to confirm this activity by performing in vitro deubiquitination assays, as no specific E3 ligase for RPS3 is available. 

Ubiquitination plays an important role in regulating protein localization [36,37], and there is a precedent for K63-linked polyubiquitination in regulating the relative cytoplasmic vs. nuclear abundance of proteins [38]. In the context of SseL, deubiquitination of K63-RPS3 significantly reduced the extent of RPS3 nuclear translocation (Figure 4). This phenotype was dependent upon SseL DUB activity, as the SseL C262A mutant had no impact on RPS3 nuclear translocation. Overall, we propose that, similarly to *E. coli*, an important aspect of *Salmonella* virulence may be that *Salmonella* injected at least one effector through their conserved T3SS into host cells where they interfere with host cell signaling cascades, particularly the RPS3 signaling, to modulate host innate immune responses to the pathogen’s advantage. 

## 4. Materials and Methods 

***Cloning, Chemicals, and Antibodies.*** The strains and plasmids used in this study are listed in Table 1. Chemicals were used according to manufacturers’ recommendations and were obtained from (Sigma Corporation: Kawasaki, Japan), except for the following: Polyjet DNA In Vitro Transfection Reagent (SignaGen Laboratories, Rockville, MD, USA), glutathione sepharose 4B (GE healthcare Life Sciences, Marlborough, MA, USA), nickel-nitrilotriacetic acid (Ni-NTA) agarose beads (Qiagen, Hilden, Germany), and TNF-α (Cell Signaling, Danvers, MA, USA). Antibodies were obtained from the following sources: anti-HA and anti-FLAG from Sigma; anti-RPS3 from Proteintech Group; anti-β–tubulin, anti-β–actin, and anti-His from Santa Cruz Biotechnology (Santa Cruz Biotechnology, Inc. Dallas, TX, USA); anti-IκBα and anti-ubiquitin from Cell Signaling; anti-PARP from BD Transduction Laboratories.

***Cell culture and transient DNA transfection.*** HEK293 cells were maintained at 37 °C, 5% CO_2_ in DMEM supplemented with 10% fetal bovine serum (FBS) and penicillin-streptomycin (100 U/mL). Cells were seeded in a 6-well plates 18–24 h prior to transfection. Media was replaced with 1 mL fresh complete DMEM per well 0.5–1 h before transfection. DNA was transfected into cells using Polyjet transfection reagent (SignaGen Laboratories). After 24 h of incubation at 37 °C, the cells were harvested.

***Cell fractionation.*** Nuclear and cytosolic protein extracts were obtained as described previously [23]. Briefly, HEK293 cells were transfected and 48 h later, TNF-α was added at 50 ng/mL for 30 min. Nuclear and cytosolic protein extracts were prepared using the NE-PER nuclear and cytoplasmic extraction reagents (Thermo Fisher: Thermo Fisher Scientific, Waltham, MA, USA). Data were analyzed by Western blotting for nuclear RPS3. Poly (ADP-ribose) polymerase and β-tubulin or β–actin were used to normalize the protein concentrations of nuclear and cytoplasmic fractions, respectively.

***Co-immunoprecipitation Assay.*** Transfected HEK293 cells were washed using pre-chilled PBS, scraped into ice-cold PBS, and centrifuged at 16,000× *g* for 5 min. Supernatants were removed, and cells were lysed in 50 mM Tris-HCl, pH 7.4, 0.15 mM NaCl, 1 mM EDTA, 1% Triton X-100, supplemented with 1× halt protease inhibitor cocktail (Thermo Fisher). Samples were incubated on ice for 30 min, with occasional shaking, and cell lysates were collected by centrifugation at 12,000*× g* for 10 min at 4 °C. Anti-FLAG M2 Gel (Sigma) was centrifuged at 7000× *g* for 30 s at 4 °C, supernatants were removed, and beads were washed twice using 50 mM Tris-HCl and 250 mM NaCl, pH 7.4 (TBS). Prepared beads were incubated with lysates for 45 min at 4 °C. The mixture was pelleted by centrifugation at 7000*× g* for 1 min at 4 °C and washed 3 times with pre-chilled TBS. The beads were resuspended in 2× SDS loading dye, incubated for 5 min at 95 °C, and analyzed using 10% SDS-PAGE.

***Protein purification.*** RPS3 was cloned into pET28a, and SseL was cloned into pET42a. They were expressed in *E. coli* BL21(DE3) cells. Bacterial cultures were grown to *A*_600_ = 0.5, and isopropyl β-d-thiogalactopyranoside (IPTG) was added to a final concentration of 0.5 mM. After 3 h of additional growth, cells were pelleted using centrifugation and lysed in 50 mM sodium phosphate, pH 8.0, 0.5 mg/mL lysozyme. Lysates were incubated on ice for 30 min with occasional shaking, after which an equal volume of 50 mM sodium phosphate, pH 8.0, 1 M NaCl, 8 mM imidazole, 20% glycerol, and 1% sarkosyl was added, followed by further incubation on ice for 30 additional minutes. The bacterial lysate was sonicated and then clarified by centrifugation. The supernatant was incubated with nickel-nitrilotriacetic acid beads (Qiagen) with end-to-end rotation for 1 h at 4 °C, and slurries were loaded on a Poly-Prep Chromatography Column (BioRad: Bio-Rad Laboratories, Hercules, CA, USA) and washed twice with 5–7 bead volumes of 50 mM sodium phosphate, pH 8.0, 600 mM NaCl, 60 mM imidazole, and 10% glycerol. Proteins were eluted in 50 mM sodium phosphate, pH 8.0, 600 mM NaCl, 250 mM imidazole, and 20% glycerol. Proteins were analyzed using 10% SDS-PAGE.

***Pulldown assays.*** GST-tagged bait proteins (10 µM) were immobilized on glutathione sepharose 4B beads (GE Healthcare) in 20 mM Tris-HCl, pH 7.9, 0.1 M NaCl, 5 mM MgCl_2_, 1 mM EDTA, 1 mM DTT, 0.2 mM PMSF, 20% glycerol, and 0.1% Nonidet P-40, supplemented with 0.33 unit/µL of DNase I and RNase A. After overnight incubation at 4 °C, the beads were incubated with His-tagged proteins (10 µM) for 1 h at 4 °C. The beads were then washed 3–4 times with 20 mM Tris-HCl, pH 7.9, 1 M NaCl, 1 mM EDTA, 1 mM DTT, 0.2 mM PMSF, 20% glycerol, and 0.1% Nonidet P-40. Proteins were eluted with 10 mM reduced glutathione and analyzed using 10% SDS-PAGE.

***Deubiquitination assays.*** HEK293 cells were transfected with FLAG-RPS3, Ubiquitin-HA, in the presence of SseL-HA or SseL(C262A). The cells were washed with pre-chilled 1× PBS, and cell pellets were lysed in 50 mM Tris-HCl, pH7.4, 0.15 mM NaCl, 1 mM EDTA, 1% Triton X-100, supplemented with 1× halt protease inhibitor cocktail (Thermo Fisher) on ice for 30 min and then mixed with anti-FLAG M2 affinity resin and rotated at 4 °C for 2 h. The resins were centrifuged at 7000× *g* for 30 s at 4 °C and then washed three times with pre-chilled TBS. The resins were resuspended in 2× SDS loading dye, boiled for 5 min at 95 °C, and immunoblotted with appropriate antibodies.

***RT-PCR***. Total RNA was isolated from cells using the RNeasy Plus Mini kit (QIAGEN). RNA was first reverse-transcribed using a first-strand cDNA synthesis kits (QIAGEN) and quantitative PCR was then carried out using a Rotor-Gene SYBR Green kit (QIAGEN). The comparative C_t_ method was used to calculate the relative abundance of IL-8 transcripts with normalization to beta-actin expression.

***Statistical analyses.*** Protein abundance was quantified using Li-COR Image Studio software. RPS3 nuclear translocation and ubiquitination were analyzed statistically using the Kruskal–Wallis test or the Dunn’s multiple comparison test where appropriate. *p*-values < 0.05 were considered significant.

## Figures and Tables

**Table 1:** Strains and plasmids used in this study.

Strain/Plasmid	Description	Source
**Strain**		
*E. coli* BL21(DE3)	*E. coli* F^−^*omp*T *hsd*SB (r_B_^−^_m _B_^−^) *gal dcm* (DE3)	Novagen
BL21(DE3)/NleH1-pET42a	GST-EHEC NleH1	[19]
BL21(DE3)/SseL-pET42a	GST-*S. Typhimurium* SseL	This study
BL21(DE3)/RPS3-pET28a	His-RPS3	This study
BL21(DE3)NleB1-pET42a	GST-EHEC NleB1	[23]
BL21(DE3)/SseL(C262A)-pET42a	GST-*S. Typhimurium* SseL(C262A)	This study
**Plasmid**		
HA	HA fusion expression	Clontech
NleH1-HA	HA fused to *E. coli* EDL933 NleH1	[19]
SseL-HA	HA fused to *S. Typhimurium* SseL	This study
SseL(C262A)-HA	HA fused to *S. Typhimurium* SseL(C262A)	This study
FliC-HA	HA fused to ETEC FliC	[39]
3× FLAG	FLAG expression	Sigma
3× FLAG-RPS3	FLAG-RPS3	[16]
pET42a	Bacterial GST fusion expression	Novagen
NleH1-pET42a	GST-EHEC NleH1	[19]
NleB1-pET42a	GST-EHEC NleB1	[23]
SseL-pET42a	GST-*S. Typhimurium* SseL	This study
SseL(C262A)-pET42a	GST-*S. Typhimurium* SseL(C262A)	This study
